# Practical Applications of Koch’s Discovery

**Published:** 1883-05

**Authors:** 


### Practical Applications of Koch's Discovery.

Dr. Gairdner, of Glasgow, is of opinion that the practical applications of Koch's discovery are to be looked for chiefly in two directions. First, in that of prevention. "This discovery," he remarked at the Glasgow Medical Society, "imposed on medical men the necessity of looking to the observance of the most scrupulous cleanliness. They must have clean hospitals, clean wards and walls, clean rooms and floors. More than ever must this now be the order of the day. They must keep in view that no man was safe unless he got everything round him as clean and as pure as could possibly be managed. Another possible direction in which what they had learned might be applied (though the question was so obscure that probably even Koch would not push it), was in making experiments to ascertain whether the tubercle-bacillus could be cultivated into a milder form. Were this possible, the question might arise whether, as in the case of small-pox and anthrax, the milder form might be utilized as a prophylactic against the more virulent form. In the matter of cure, too, attempts must be made to apply the discovery. For the next year or two, there would be a run on the indiscriminate use of antiseptics in the treatment of phthisis; and probably this would result in disappointment. But this ought not to discourage them, as they might feel sure that, whatever residuum of utility there existed in the antiseptic treatment of phthisis, would eventually be made clear."—*British Medical Journal.*

PROF. VERNEUIL says that when a person has at any previous time suffered from an attack of erysipelas he is much more liable, in case he is wounded, to contract traumatic erysipelas than a patient who has been free from the disease.—*Gazette de Med. de Paris.*

Probably the smallest abrasions, as those of the lips and face, may open the gates to the admission of erysipelas in one who has previously had it.

Here is a caution to the dentist.—ED.

